# Postoperative radiotherapy improves local control and survival in patients with uterine leiomyosarcoma

**DOI:** 10.1186/1748-717X-8-128

**Published:** 2013-05-24

**Authors:** Philip Wong, Kathy Han, Jenna Sykes, Charles Catton, Stephane Laframboise, Anthony Fyles, Lee Manchul, Wilfred Levin, Michael Milosevic

**Affiliations:** 1 Radiation Medicine Program, Princess Margaret Cancer Centre, University of Toronto, 610 University Avenue, Toronto, ON M5G 2M9, Canada; 2 Department of Radiation Oncology, University of Toronto, Fitzgerald Building, 150 College Street, Toronto, ON M5S 3E2, Canada; 3 Department of Biostatistics, Princess Margaret Cancer Centre, University of Toronto, 610 University Avenue, Toronto, ON M5G 2M9, Canada; 4 Department of Gynaecologic Oncology, Princess Margaret Cancer Centre, University of Toronto, 610 University Avenue, Toronto, ON M5G 2M9, Canada; 5 Radiation Medicine Program, Princess Margaret Cancer Centre, 5th Floor Rm 985, 610 University Avenue, Toronto, ON M5G 2M9, Canada

**Keywords:** Radiotherapy, Uterine, Leiomyosarcoma, Competing risk, Recurrence, Survival

## Abstract

**Background:**

To examine the role of radiotherapy (RT) in uterine leiomyosarcomas (LMS) and to determine the patient population who may benefit from RT.

**Methods:**

From 1998–2008, 69 patients with primary uterine LMS underwent hysterectomy with or without pelvic radiotherapy to a median dose of 45 Gy. Univariate analysis was performed using the Kaplan-Meier method and cumulative-incidence function, and multivariate analyses using Fine and Gray or Cox proportional hazard models.

**Results:**

Following surgery, 32 out of 69 patients received RT. There was no evidence of any correlation between patient, disease and treatment characteristics and the use of RT. Median follow-up was 57 months. RT was associated with reduced local recurrence (3y LR 19% vs. 39%; Gray’s p = 0.019) and improved overall survival (3y OS 69% vs. 35%; log-rank p = 0.025) on univariate analysis. Multivariate analysis demonstrated that RT reduced LR (HR: 0.28, CI: 0.11-0.69, p = 0.006) and increased OS (HR: 0.44, CI: 0.23-0.85, p = 0.014) independent of other clinical and pathologic factors. Positive surgical margins increased the odds of LR (HR: 5.6, CI: 2.3-13.4, p = 0.00012). Large tumor size and advanced stage (II-IV) were associated with the development of distant metastases and inferior OS.

**Conclusions:**

Postoperative pelvic RT reduces LR and improves OS of patients with uterine LMS.

## Background

Uterine leiomyosarcoma (LMS) is the most common sarcoma arising from the uterus and comprises approximately 2% of uterine cancers [1]. Patients diagnosed with LMS have a 5-year overall survival (OS) ranging from 25-75% [1-3]. The primary management of LMS is operative. Although earlier reports found benefits to adding adjuvant radiotherapy (RT) in the management of uterine sarcomas, these studies included carcinosarcomas or malignant mixed müllerian tumor which are now re-categorized as a metaplastic carcinoma that predominantly recur locally, unlike sarcomas [1]. Three recent retrospective studies that investigated the use of RT specifically in LMS yielded mixed results [2,4-6]. Giuntoli et al. reviewed the outcomes of 208 LMS, in which 31 patients received RT. In their analysis, RT significantly improved local control and also trended (p = 0.056) towards better disease-specific survival on multivariate analysis [2]. Similarly, Mahdavi et al. observed an improved local control (p = 0.02) in the 24 LMS patients who received RT compared to 123 who did not receive RT [5]. Finally, Garg et al. extracted data from the Surveillance, Epidemiology, and End Result database between 1988 and 2005 and identified 819 patients with stage I (FIGO 2009) LMS, of whom 201 received RT [4]. There was no survival advantage with RT but patients in the RT cohort had more advanced disease at diagnosis.

The European Organization for Research and Treatment of Cancer (EORTC) conducted a randomized trial to investigate the efficacy of RT in high grade uterine sarcomas [6]. This study accrued 224 patients with carcinosarcoma, LMS or endometrial stromal cancers from 36 institutions over a 13-year period. Though patients were prospectively stratified by pathologic diagnosis, the study was not powered to investigate the use of RT in individual pathological groups. On subgroup analysis of the 99 patients with LMS, although there were less local recurrences in patients who received RT (20% vs. 32%), RT did not significantly improve local control or survival.

Prior studies focusing on LMS have reported several clinical and pathological characteristics to be prognostic for clinical outcomes. Specifically, advanced age (>55 years), high tumor grade, larger tumor size (>5 cm), high mitotic index (≥ 15 per high power field), and omission of bilateral oophrectomies have been correlated with worse outcomes [4,5,7,8]. In addition to the above mentioned factors, Park et al. recently observed that tumor morcellation [9] increases local recurrence in the abdomen and pelvis (44% vs. 13%) and was associated with shorter survival (hazard ratio (HR):3.1 (p = 0.038)).

The current study examines the role of RT in patients with uterine LMS treated at the Princess Margaret Cancer Centre and to determine potential sub-groups who may benefit from RT.

## Methods

### Patient data collection

Research Ethics Board approval from the University Health Network (UHN) in Toronto was obtained. Adult patients who were treated with a hysterectomy for primary uterine LMS’s from January 1998 until December 2008 were identified using an institutional cancer registry and radiation oncology electronic patient database. Endometrial stromal sarcomas and carcinosarcomas (malignant mixed mullerian tumors) were excluded. Sixty-nine patients fulfilled the above criteria and their medical records were reviewed.

### Treatment methods

All patients underwent hysterectomy. Histology was reviewed at the UHN prior to treatment if surgery was performed in a non-affiliated institution. More than 70% of the patients underwent hysterectomy and bilateral salpingo-oophrectomy (Table 1). Of the study’s 69 patients, 66 had a chest X-ray or CT scan during staging work-up. All 32 patients who received RT also underwent local (CT of the abdomen and pelvis or MRI of the pelvis) restaging prior to commencing RT. For those who received RT, a median dose of 45 Gray (Gy) in 25 daily fractions was given to the whole pelvis using high energy (18–25 MV) beams and either a 4-field (n = 24) or parallel opposed (n = 8) field arrangement. The use of postoperative chemotherapy (n = 9) was determined by individual treating medical oncologists.

**Table 1:** Patient characteristics

	**No RT n = 37 (%)**	**RT n = 32 (%)**	**p-value**
**Margin**			
Negative (n = 49)	27 (73%)	22 (69%)	Fisher’s exact test p = 0.99
Positive (n = 19)	10 (27%)	9 (28%)	
Unknown (n = 1)	0 (0%)	1 (3%)	
**Size (median)**	9.0	10.0	*t*-test p = 0.83
**Age (median)**	54.7	51.5	*t*-test p = 0.65
**Grade**			
High (n = 64)	33 (89%)	31 (97%)	Fisher’s exact test p = 0.36
Low (n = 5)	4 (11%)	1 (3%)	
**Figo 2009 Stage**			
I (n = 35)	20 (54%)	15 (47%)	*χ*^2^ test for trend in proportions p = 0.86
II (n = 20)	8 (22%)	12 (37%)	
III (n = 2)	2 (5%)	0 (0%)	
IV (n = 12)	7 (19%)	5 (16%)	
**Figo 2009 dichotomized**			
Stage I (n = 35)	20 (54%)	15 (47%)	Fisher’s exact test p = 0.63
Stage II – IV (n = 34)	17 (46%)	17 (53%)	
**Adjuvant Chemotherapy**			
Yes (n = 9)	6 (16%)	3 (9%)	Fisher’s exact test p = 0.49
No (n = 60)	31 (84%)	29 (91%)	
**Surgery Center**			
Center (n = 25)	14 (38%)	11 (34%)	Fisher’s exact test p = 0.81
Community (n = 44)	23 (62%)	21 (66%)	
**Bilateral oophrectomy**			Fisher’s exact test p = 0.57
Yes	29 (78%)	23 (72%)	
No	7 (19%)	8 (25%)	
Unknown	1 (3%)	1 (3%)	
**Surgery**			Fisher’s exact test p = 0.49
Subtotal Hysterectomy	6	3	
Total Hysterectomy	31	26	
Radical Hysterectomy	0	2	
Exenteration with ileal conduit	0	1	

### Statistical analysis

The following variables were collected and included in the analysis: Patient age at diagnosis, surgical margin status, pathological maximum tumor size, grade (Low: Broders grade 1 vs. High), site of surgery (Community Vs. Academic), stage (FIGO 2009 system [10]), and use of postoperative chemotherapy. Surgical margins were categorized as “negative” or “positive”. The tumor margins were considered “positive” if there was tumor morcellation, tumor spillage or the closest pathologic margin was <1 mm without a fascia/serosa boundary [11].

The Fisher’s exact test was used to determine if there were differences in the distribution of categorical variables between the two treatment groups (RT vs. no RT). The student *t*-test was used for continuous variables. The primary outcome of interest was local recurrence (LR) and secondary outcomes were distant metastases (DM), disease-free survival (DFS) and overall survival (OS). LR was defined as recurrence within the pelvis, DM as recurrence beyond the pelvis, DFS as freedom from any recurrence or death and OS as freedom from death of any cause. LR and DM were considered synchronous if the events were detected less than 2.5 weeks apart. Time to event was defined as the time from the date of diagnosis to the date of the event, or date of last follow-up. For DFS and OS, three- and five-year survival probabilities were generated using the Kaplan-Meier method and differences in survival curves were compared using the log-rank test. The effect of RT on OS and DFS was also examined using Cox proportional hazards models while adjusting for clinical factors found significant on univariate analysis and performing a backwards selection. For LR and DM, univariate analysis was performed using cumulative incidence and Gray’s test to account for competing risks from relapse at a different site and death without relapse. Multivariate hazard ratios (HRs) were reported as subdistribution hazards as outlined by the Fine and Gray model [12]. Proportional hazards assumptions were checked for all models using scaled Schoenfeld residuals.

Acute and chronic toxicities were defined as toxicities related to RT occurring within or beyond 90 days of RT completion respectively, and graded using the Common Terminology Criteria for Adverse Events v4.0.

All analyses were done using R version 2.12.1 and SAS version 9.2. A two-sided p-value of 0.05 was used to assess statistical significance.

## Results and discussion

### Patient characteristics

From 1998–2008, 69 patients with uterine LMS were treated. Following surgery, 32 patients received RT. No differences were found between patient, disease and treatment characteristics and those who did and did not receive RT (Table 1). 51% of the patients had FIGO 2009 stage I disease. 71% of the patients had negative surgical margins. The proportions of tumors morcellated (n = 10) or removed with microscopic (n = 8) or macroscopic positive margins (n = 1) were similar in both cohorts (Fisher exact test p = 0.169). Median follow-up was 57 (range: 1.7-149.7) months for alive patients.

### Local recurrence

**Figure 1:**
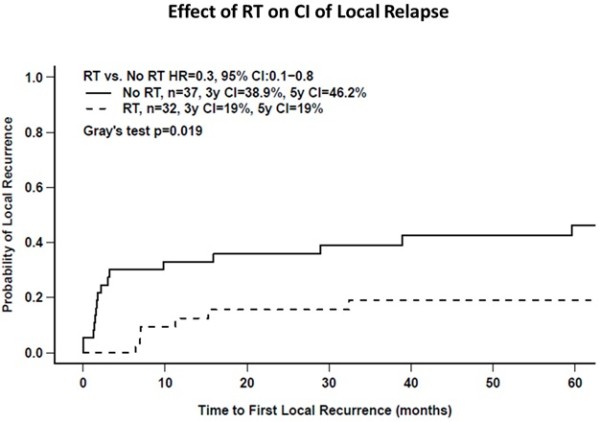
Cumulative incidence of local relapse as a function of the use of postoperative radiotherapy (RT).

There were 22 LRs that occurred prior to any other relapse or deaths. The overall 3-year cumulative incidence of LR was 30%. On univariate analysis, there were fewer LRs in patients who received pelvic RT compared to those treated with surgery alone (3y-LR 19% vs. 39%, Gray’s p = 0.019) as illustrated in Figure 1 and Table 2. Patients with large tumor size, more advanced stage disease, positive surgical margin or who received postoperative chemotherapy also were more likely to experience LR. Cox multivariate analysis demonstrated that RT was associated with decreased LR independent of other clinical and pathologic factors (HR:0.28, 95% CI: 0.11-0.69, p = 0.006) (Table 3). Surgical margin status was the only other factor independently associated with LR (HR:5.6, 95% CI:2.3-13.4, p = 0.00012).

**Table 2:** Univariate analysis of the effect of adjuvant radiotherapy (RT) and other variables on local relapse, distant relapse, disease-free survival and overall survival

**Variables**	**Local relapse**	**Distant relapse**	**Disease-free survival**	**Overall survival**
	**HR**	**HR**	**HR**	**HR**
	**(95% CI, p-Value)**	**(95% CI, p-Value)**	**(95% CI, p-Value)**	**(95% CI, p-Value)**
**RT (RT vs. No RT)**	0.3	1.6	0.7	0.5
	(0.1–0.8, 0.019)	(0.9–3.0, 0.12)	(0.4–1.1, 0.11)	(0.3–0.9, 0.028)
**Margin (Positive vs. Negative)**	4.6	NS	2.8	3.0
	(2.0–10.7, 0.0004)		(1.5–5.0, 0.00082)	(1.6–5.7, 0.00061)
**Size (≥9 cm vs. <9 cm)**	3.0	2.6	2.9	3.6
	(1.2–7.5, 0.021)	(1.4–4.9, 0.0023)	(1.6–5.1, 0.00023)	(1.8–6.9, 0.00016)
**Age (>51 vs. ≤51)**	NS	NS	NS	NS
**FIGO 2008 Stage (II–IV vs. I)**	2.7	2.3	3.1	3.6
	(1.1–6.4, 0.025)	(1.3–4.3, 0.0048)	(1.7–5.3, <0.0001)	(1.8–6.9, 0.00019)
**Adjuvant Chemo (Yes vs. No)**	8.3	NS	4.2	6.9
	(3.4–20.6, <0.0001)		(2.0–9.1, 0.0099)	(2.9–16.3, <0.0001)
**Surgery Center (Community vs. Academic)**	NS	NS	NS	NS

Values HR (p-value).

**Table 3:** Multivariate Cox proportional hazards for local relapse, distant relapse, disease-free survival and overall survival

**Hazard Ratio**				
**Variable**	**Local recurrence (LR)**	**95% CI**		**p-value**
RT (Yes vs. No)	0.3	0.1	0.7	0.006
Margins (Neg vs. Pos)	5.6	2.3	13	0.00012
	**Distant recurrence (DM)**			
RT (Yes vs No)^a^	0.2	0.06	0.7	0.0087
RT over log time^b^	2.9	1.6	5.5	0.00064
Stage (2–4 vs 1)	2.3	1.3	4.3	0.0069
	**Disease free survival (DFS)**			
RT (Yes vs No)^a^	0.1	0.04	0.5	0.0015
RT over Log time^b^	1.9	1.2	3.2	0.012
Log Size	2.1	1.2	3.7	0.011
Stage (2–4 vs 1)	3.6	1.9	6.6	<0.0001
	**Overall survival (OS)**			
RT (Yes vs No)	0.4	0.2	0.8	0.014
Size	1.1	1.1	1.2	<0.0001
Stage (2–4 vs 1)	3.0	1.4	6.3	0.004

^a^ Effect of RT on outcome (DM or DFS) at the completion of treatment.

^b^ RT logarithmic time-dependent coefficient.

### Distant metastases

**Figure 2:**
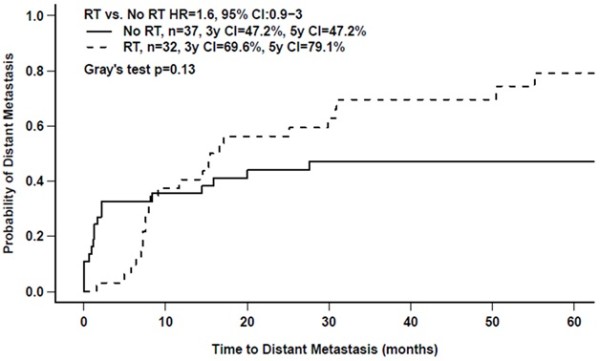
Cumulative incidence of distant relapse as a function of the use of postoperative radiotherapy (RT).

Forty-three DMs occurred prior to any other relapse or deaths. The overall 3-year cumulative incidence of DM was 58%. On univariate analysis, patients with a large tumor or advanced stage disease were significantly more likely to develop distant relapses (Table 2). On Cox multivariate analysis, the assumption of proportional hazard between the use of postoperative RT and DM was violated, implying that the effect of RT was not constant over time after diagnosis. To correct for this, a statistical interaction term between RT and a logarithmic function of time was included in the Cox model. Immediately after diagnosis, RT was associated with a significantly lower risk of DM (HR:0.19, 95% CI:0.055-0.66, p = 0087, Table 3) independent of other clinical and pathologic factors. However, this effect diminished with increasing follow-up (HR:2.9, 95% CI:1.6-5.5, p = 0.00064, Table 3) and was lost by about 10 months as shown in Figure 2.

### Disease-free survival

**Figure 3:**
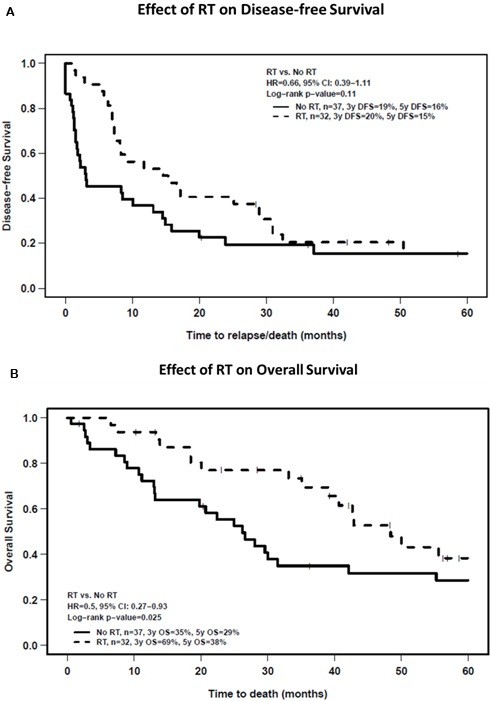
Kaplan curves of A) disease-free survival and B) overall survival as a function of the use of postoperative radiotherapy (RT).

There were 58 recurrences or deaths. Median DFS time was 8.5 (95% CI:7–16) months. On univariate analysis, DFS was significantly worse in the setting of large tumor size, advanced stage, positive margins or the use of postoperative chemotherapy (Table 2). RT had no apparent effect on DFS (Figure 3A). However, similar to the result for DM, the effect of RT was not constant over time. When this was accounted for using time-varying coefficients in the Cox multivariate analysis, RT was found to have a significant, independent effect on DFS early after diagnosis (HR:0.14, 95% CI:0.044-0.48, p = 0.015, Table 3) that diminished with increasing follow-up (HR:1.9, 95% CI:1.2-3.2, p = 0.011, Table 3). Tumor size and stage also were independently associated with DFS.

### Overall survival

There were 44 deaths and the median OS time was 39 (95% CI:28–56) months. On univariate analysis (Table 2), patients undergoing RT survived longer than patients without RT (3y-OS:69% vs. 35%; log-rank p = 0.025) (Figure 3B). Large tumor size, advanced stage, a positive surgical margin or the use of chemotherapy was associated with worse OS. Multivariate analysis (Table 3) demonstrated that RT significantly improved OS (HR:0.44, 95% CI: 0.23-0.85, p = 0.014) independent of other clinical and pathologic factors.

### Toxicity

Acute toxicities secondary to RT occurred in 24 of the 32 patients: 16 (50%) of the patients had Grade 1 or 2 gastrointestinal (GI) toxicities, 2 (6%) patients had Grade 3 GI toxicities (diarrhea). Four (12%) patients had Grade 1 or 2 genitourinary (GU) acute toxicities. Three (9%) patients had chronic side-effects related to RT: 1 Grade 1 (GU) and 2 Grade 2 (GU).

### Discussion

Uterine LMS is rare but aggressive, with a high propensity to metastasize [1-3]. Despite the importance of systemic disease in causing patient death, local recurrences are also prevalent and may be a competitive cause of patient death and suffering [2,13-15]. In the current study, the 3y DFS was 20% and the cumulative incidence rates for LR and DM as the first sites of relapse were 30% and 58% respectively. The 3y OS was 51%. These findings are comparable to previous reports [7] and highlight the need to improve both local and systemic disease control. The use of postoperative pelvic RT reduced LR and DM although, for the latter, only in the interval shortly after completing treatment. These reductions in LR and DM translated to improved OS.

Although there are reports of improved local control with pelvic RT in patients with uterine LMS [2,5], there was no apparent survival benefit in the previously described EORTC 55874 randomized trial [6] despite the improved local control in patients who received postoperative RT. The EORTC study recruited only patients with FIGO 1998 stage I-II disease that had been completely removed. Since the publication of the EORTC results, a new FIGO staging system has been developed (FIGO 2009) specifically for LMS [10] and all patients in the EORTC study would be classified as having stage I disease under the new system. In contrast, in the current study, only 51% of patients were stage I with the rest having more advanced disease and therefore potentially more likely to benefit from the addition of pelvic RT.

This study sheds new light on the relationship between pelvic RT and the pattern of metastatic failure in patients with uterine LMS. Using a competing risk multivariate model (Table 3), pelvic RT was found to be associated with a significant reduction in the risk of DM early after diagnosis and treatment but this benefit diminished with time and was lost by 10 months (Figure 2). To some extent, this may be an artefact of how recurrences were classified (site of first recurrence as local or metastatic) and the competing risk methodology. However, it also is compatible with RT improving local control, thereby allowing occult micro-metastases to grow and become clinically detectable in patients who otherwise would have died sooner of progressive pelvic disease. An important beneficial effect of pelvic RT on overall patient outcome is supported by the fact that pelvic RT was associated with a 2.3-fold improvement in survival independent of other important prognostic factors (Figure 3b and Table 3). Regardless, all patients with uterine LMS are at high risk of developing DM and more effective forms of systemic treatment are needed to mitigate this risk. As patient with RT increasingly developed DM later, the rate of DFS and OS of these patients are reduced and converge with patients who did not receive RT by year-3 (DFS) and year-5 (OS).

Examining patients with all stages of uterine LMS permitted the identification of patients who would most likely benefit from the addition of pelvic RT as those who had a large tumor, advanced stage or a positive surgical margin (Tables 2 and 3). A positive margin was associated with local recurrence, whereas large size and advanced stage predisposed to the development of DM and reduced survival. For the purpose of this analysis, a positive margin was defined by tumor morcellation, intraoperative tumor spillage or a close resection margin <1 mm without a fascia/serosa boundary. This definition was derived from the International Union Against Cancer [16] and Enneking [11] margin definitions used for soft-tissue sarcomas arising at other sites, in which patients with microscopically positive margins are at 3–4 times higher risk for LR [17-19]. In line with these studies, Park et al. recently demonstrated that tumor morcellation in LMS increased the rate of abdomino-pelvic recurrence by a factor of 3.4, and worsened DFS and OS [9]. In the current study, LR was 5.6-fold more likely in patients with a positive surgical margin according to this definition.

Like other retrospective studies, the current analysis may be affected by sample size and power limitations, incomplete information, referral and researcher biases, as well as unbeknownst confounding factors. The use of postoperative RT was not randomly assigned but rather determined by the treating physicians. To account for bias in this decision, a large number of patient, disease and treatment variables were collected to ensure that the groups were characterized as fully as possible for known prognostic factors. The potential confounding role of postoperative chemotherapy is limited as small number (9) of patients received chemotherapy and this factor was included in the multivariate analysis. Additional univariate analysis on the effect of RT sub-categorized by stage of disease (I/II vs. III/IV) demonstrated similar trends for OS, DFS and LC, suggesting the effects of RT is present in early and late stages. Researcher biases were limited by employing two authors to independently review the data of patients who did or did not receive RT. Criteria for categorization of each of the variables were predetermined prior to data collection. As all patients in the study were treated at a tertiary academic center, the possibility of a referral bias for more aggressive disease cannot be excluded. Nevertheless, two-thirds of the patients were operated in a community center. No difference in outcome was observed between patients who underwent surgery in an academic hospital vs. those who had surgery in a community hospital (Table 2).

## Conclusions

In summary, a positive surgical margin significantly increases the risk of LR in patients with uterine LMS and pelvic RT reduces this risk leading to an improvement in OS. Positive margins by the definition used in this study may be more common when a diagnosis of LMS is established unexpectedly during or after an operation for a presumed leiomyoma, and/or in patients with locally extensive LMS. Although LMS’s are rare in relation to leiomyomas, pre-operative planning to avoid morcellation and positive surgical margins may potentially improve local control. Significant morbidities and deaths secondary to pelvic LR may be reduced by optimizing surgery and RT. Finally, despite RT improving the local control and OS of patients with LMS, a large proportion of surviving patients subsequently develop DM, thus highlighting the importance of discovering new and efficacious strategies against systemic mestastases.

## Competing interests

Philip Wong and Kathy Han are both recipients of the CARO-Elekta research fellowships. There are no other conflicts of interests from the other authors.

## Authors’ contributions

PW, KH, JS, CC and MM contributed to conception and design of the study. PW, KH, JS and MM acquired, analysed and interpreted the data. All authors (PW, KH, JS, CC, SL, AF, LM, WL and MM) were involved in the critical appraisal, drafting, and revising of the manuscript. All authors read and approved the final manuscript.
